# Supplementary material for: Structural Connectivity Alterations in Operculo-Insular Epilepsy
Source: Brain Sci. 2021 Aug 5;11(8):1041. doi: 10.3390/brainsci11081041 (PMC8392362; doi:10.3390/brainsci11081041)
Supplement: Supplementary file 1 [file brainsci-11-01041-s001.zip › Supplementary Materials.pdf]

Supplementary Material

Supplementary Figures

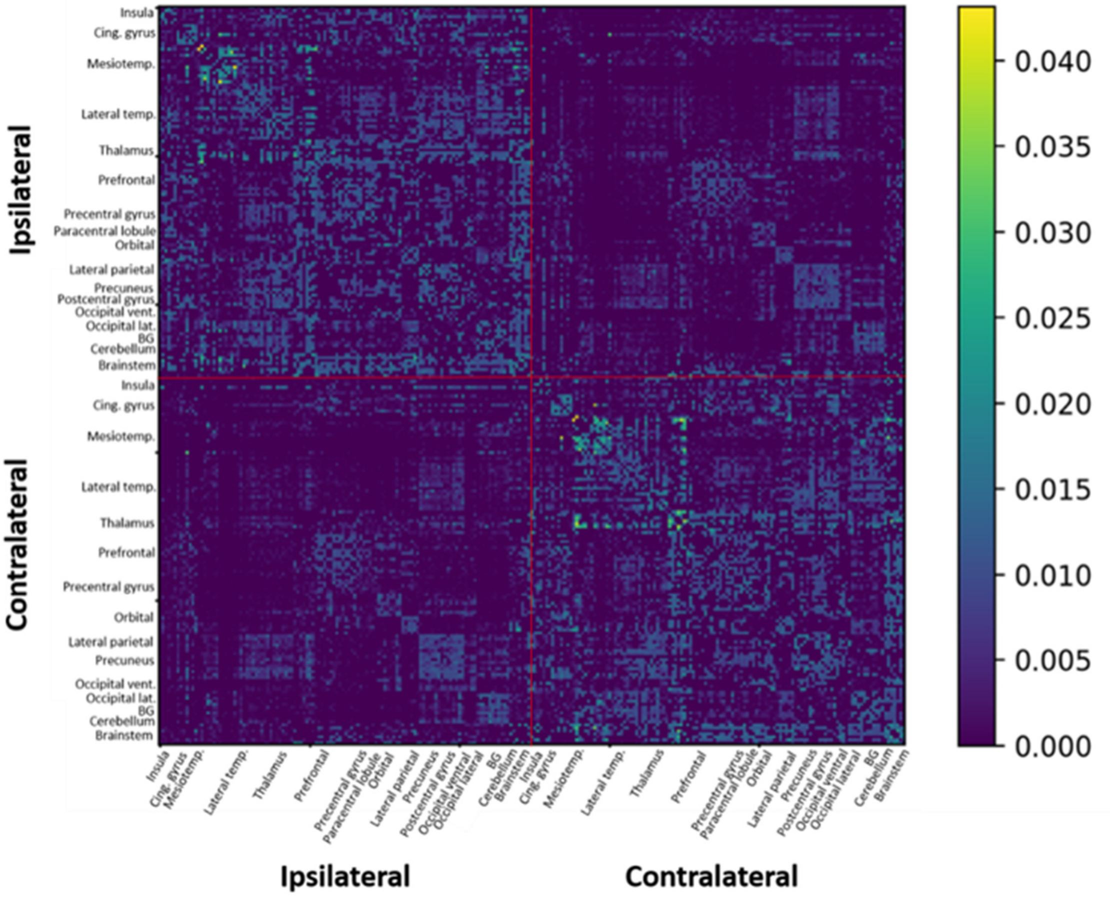

Figure S1. Average whole-brain COMMIT-weighted connectivity matrix in the OIE group. The matrix was masked based on a similarity threshold calculated in HCs. The colour bar corresponds to the measured COMMIT weight.

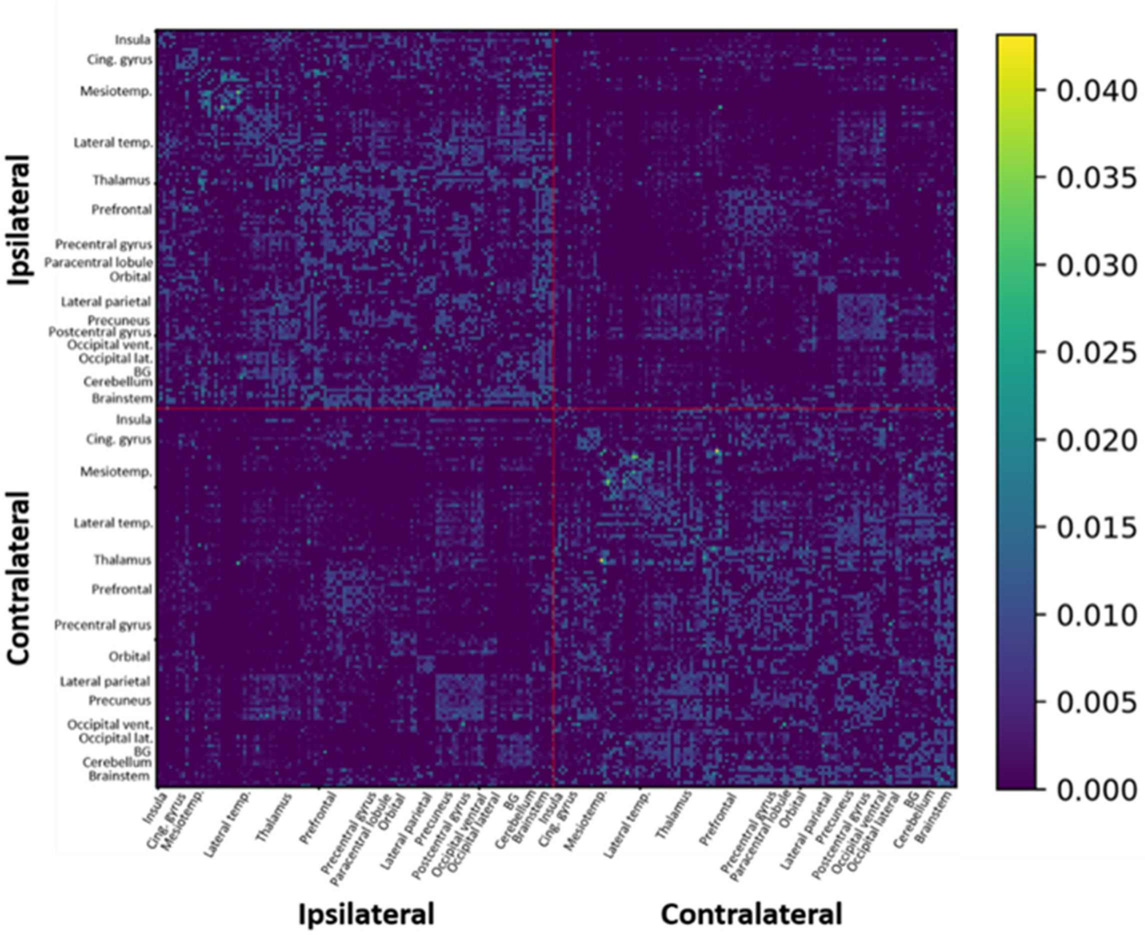

Figure S2. Average whole-brain COMMIT-weighted connectivity matrix in the TLE group. The matrix was masked based on a similarity threshold calculated in HCs. The colour bar corresponds to the measured COMMIT weight.

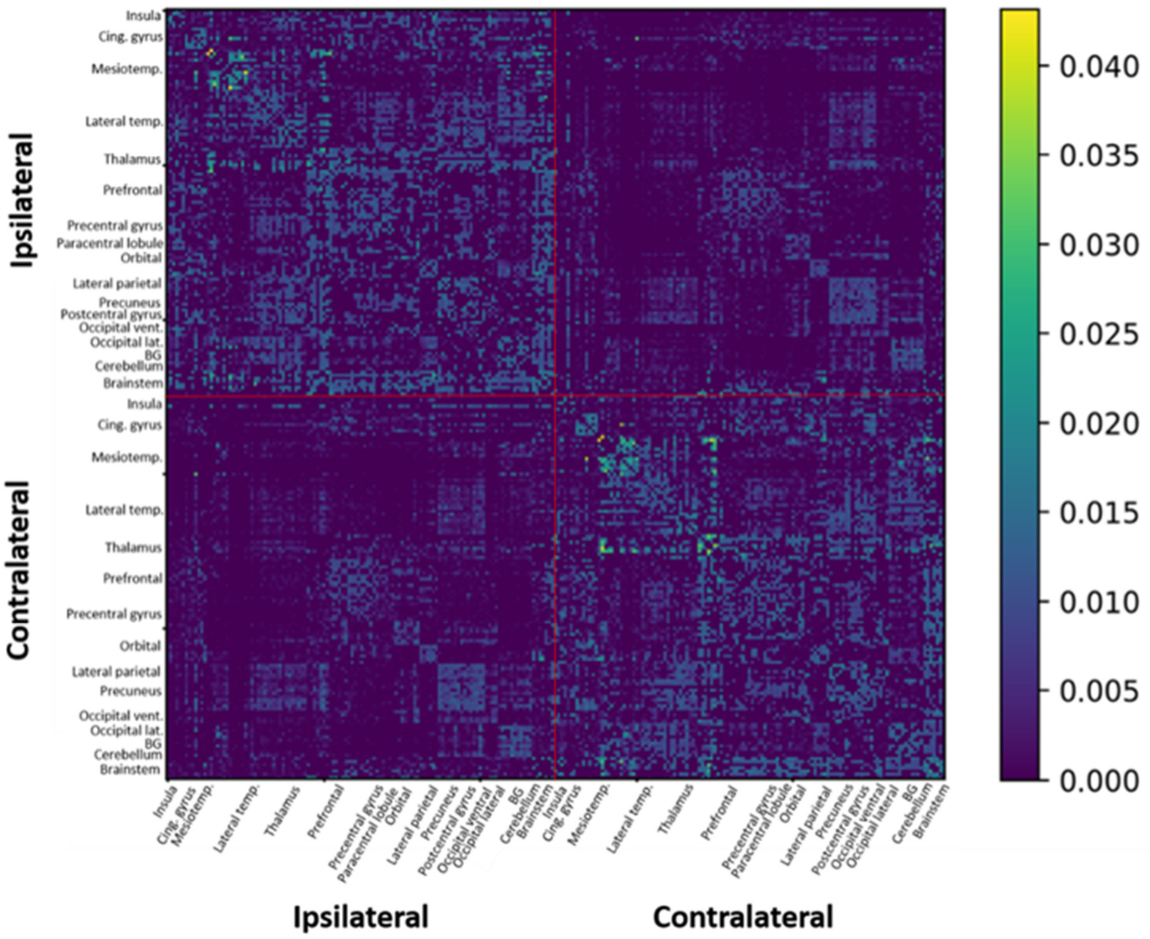

Figure S3. Average whole-brain COMMIT-weighted connectivity matrix in the HC group. The matrix was masked based on a similarity threshold calculated in HCs. The colour bar corresponds to the measured COMMIT weight.

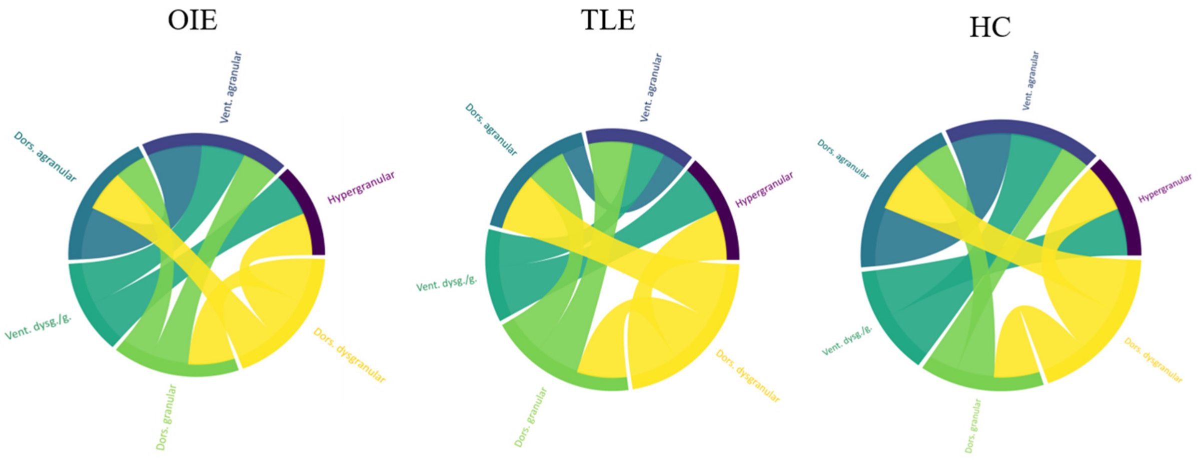

Figure S4. Average COMMIT-weighted connectome rings of the insular subnetwork in OIE, TLE and HC participants. The submatrix was masked based on a similarity threshold calculated from the insular submatrices of HCs. The width of the colored connections is proportional to the COMMIT weight of the connection. Vent.= ventral; Dors.= dorsal; dysg./g. = dysgranular/granular.

Supplementary Tables

**Reduced connectivity in patients with OIE**		
Ipsilateral orbital gyrus	-	Ipsilateral lateral occipital cortex (V5_MT)
Ipsilateral rostral MTG	-	Ipsilateral dorsal granular insula
Ipsilateral ITG (intermediate)	-	Ipsilateral globus pallidus
Ipsilateral frontal operculum	-	Ipsilateral rostral hippocampus
Contralateral caudodorsal cingulate gyrus	-	Contralateral ventral cingulate gyrus
Contralateral medial prefrontal thalamus	-	Brainstem
**Increased connectivity in patients with OIE**		
Ipsilateral rostrodorsal IPL	-	Ipsilateral lateral posterior PHG
Ipsilateral precuneus (medial area 7)	-	Ipsilateral rostral PHG
Ipsilateral precuneus (medial area 5)	-	Brainstem
Ipsilateral dorsomedial parieto-occipital sulcus	-	Ipsilateral pregenual cingulate gyrus
Ipsilateral postcentral (area2)	-	Ipsilateral ventromedial putamen
Ipsilateral dorsal granular insula	-	Ipsilateral pregenual cingulate gyrus
Ipsilateral lateral prefrontal thalamus	-	Ipsilateral MFG (inferior frontal junction)
Ipsilateral lateral prefrontal thalamus	-	Ipsilateral rostral lingual gyrus
Ipsilateral ventrolateral caudal precentral gyrus	-	Contralateral posterior parietal thalamus
Ipsilateral rostroposterior superior temporal sulvus	-	Contralateral rostroventral IPL
Ipsilateral precuneus (medial area 7)	-	Contralateral rostroposterior superior temporal sulcus
Ipsilateral dorsal agranular insula	-	Contralateral dorsolateral putamen
Ipsilateral sensory thalamus	-	Contralateral ITG (intermediate lateral area)
Contralateral lateral occipital cortex (polar cortex)	-	Contralateral postcentral gyrus (trunk region)
Contralateral lateral occipital cortex (polar cortex)	-	Contralateral dorsal cingulate gyrus
Contralateral lateral occipital (inferior occipital cortex)	-	Contralateral lateral occipital cortex (middle occipital gyrus)

Table S1. Links showing changes in COMMIT weights in OIE patients compared to HCs. Comparisons were performed using general linear models. Significance was thresholded at $p<0.001$ uncorrected. MTG = middle temporal gyrus; ITG=inferior temporal gyrus; IPL = inferior parietal lobule; PHG= parahippocampal gyrus.

**Increased connectivity in patients with OIE**		
Ipsilateral rostral PHG	-	Ipsilateral precuneus (medial area 7)
Ipsilateral rostrocaudal IPL	-	Ipsilateral caudodorsal cingulate gyrus
Ipsilateral lateral amygdala	-	Ipsilateral rostrodorsal IPL
Ipsilateral premotor thalamus	-	Ipsilateral inferior frontal sulcus
Ipsilateral rostral temporal thalamus	-	Ipsilateral lateral occipital cortex (inferior occipital cortex)
Ipsilateral caudal SPL	-	Contralateral caudal temporal thalamus
Brainstem	-	Contralateral occipital thalamus
Contralateral precentral gyrus (caudal dorsolateral)	-	Contralateral caudal temporal thalamus
Contralateral rostroventral fusiform gyrus	-	Contralateral lateral occipital cortex (middle occipital gyrus)
Contralateral lateral posterior PHG	-	Contralateral rostrodorsal IPL
Contralateral lateral occipital cortex (V5_MT)	-	Contralateral precentral gyrus (tongue and larynx regions)
Contralateral ventromedial putamen	-	Contralateral lateral SPL
Contralateral occipital thalamus	-	Contralateral lateroventral fusiform gyrus
Contralateral temporal thalamus	-	Contralateral lateral occipital cortex (V5_MT)
**Increased connectivity in patients with TLE**		
Ipsilateral medial prefrontal thalamus	-	Ipsilateral caudal medioventral occipital cortex
Ipsilateral rostral temporal thalamus	-	Ipsilateral rostral hippocampus
Ipsilateral medial STG	-	Contralateral rostral hippocampus
Ipsilateral precuneus (dorsomedial p-o sulcus)	-	Contralateral rostroposterior superior temporal sulcus
Ipsilateral medial prefrontal thalamus	-	Contralateral medial orbital
Contralateral caudal STG	-	Contralateral lateral occipital cortex (lateral superior occipital gyrus)
Contralateral dorsolateral MTG	-	Contralateral ventral agranular insula

Table S2. Links showing changes in COMMIT weights when comparing OIE to TLE patients. Comparisons were performed using general linear models. Significance was thresholded at $p<0.001$ uncorrected. PHG = parahippocampal gyrus; IPL= inferior parietal lobule; SPL = superior parietal lobule; p-o = parieto-occipital; STG = superior temporal gyrus; p-o = parieto-occipital; MTG = middle temporal gyrus.

Increased degree in patients with OIE	Increased degree in patients with TLE
Ipsilateral lateral orbital gyrus	Ipsilateral dorsal IFG
Ipsilateral paracentral lobule	Ipsilateral rostral IFG
Ipsilateral medioventral fusiform gyrus	Ipsilateral precuneus (area 3)
Ipsilateral caudal PHG	Ipsilateral lateral occipital cortex (V5_MT)
Ipsilasteral posterior PHG	Ipsilateral lateral superior occipital gyrus
Ipsilateral postcentral gyrus (area 2)	Ipsilateral medial prefrontal thalamus
Ipsilateral hypergranular insula	Contralteral medial SFG
Ipsilateral ventral agranular insula	Contralateral dorsal MFG
Ipsilateral dorsal agranular insula	Contralateral caudal SPL
Ipsilateral ventral dysgranular/granular insula	Contralateral caudal IPL
Ipsilateral dorsal granular insula	Contralateral caudal cingulate gyrus
Ipsilateral dorsal dysgranular insula	Contralateral caudal lingual gyrus
Ipsilateral rostroventral cingulate gyrus	Contralateral caudal cuneus
Ipsilateral caudal cingulate gyrus	Contralateral occipital polar cortex
Ipsilateral caudal lingual gyrus	Contralateral inferior occipital cortex
Ipsilateral rostral lingual gyrus	Contralateral caudal hippocampus
Ipsilateral occipital polar cortex	Contralateral dorsal caudate nucleus
Ipsilateral caudal hippocampus	Contralateral sensory thalamus
Ipsilateral sensory thalamus	Contralateral rostral temporal thalamus
Contralateral lateral orbital gyrus	Contralateral caudal temporal thalamus
Contralateral rostroventral fusiform	
Contralateral medioventral fusiform	
Contralateral postcentral gyrus (trunk region)	
Contralateral caudal cuneus	
Contralateral lateral occipital cortex (V5_MT)	
Contralateral inferior occipital cortex	
Contralateral lateral superior occipital gyrus	
Contralateral dorsal caudate nucleus	
Contralateral medial prefrontal thalamus	
Contralateral sensory thalamus	
Contralateral rostral temporal thalamus	
Contralateral caudal temporal thalamus	

Table S3. Changes in nodal degrees when comparing OIE to TLE patients. Comparisons were performed using two-tailed t-tests. Significance was thresholded at $p<0.05$ uncorrected. PHG = parahippocampal gyrus; IFG = inferior frontal gyrus; SFG = superior frontal gyrus; MFG = middle frontal gyrus; SPL = superior parietal lobule; IPL = inferior parietal lobule.

Increased betweenness centrality in patients with OIE	Increased betweenness centrality in patients with TLE
Ipsilateral lateral orbital gyrus	Ipsilateral lateral superior occipital gyrus
Ipsilateral precentral gyrus (trunk region)	Ipsilateral medial prefrontal thalamus
Ipsilateral paracentral lobule	Ipsilateral dorsal IFG
Ipsilateral lateral posterior PHG	Contralateral caudal lingual gyrus
Ipsilateral postcentral gyrus (upper limb, head and face regions)	Contralateral caudal cuneus
Ipsilateral postcentral gyrus (area 2)	Contralateral occipital polar cortex
Ipsilateral hypergranular insula	Contralateral caudal hippocampus
Ipsilateral ventral agranluar insula	Contralateral dorsal caudate nucleus
Ipsilateral dorsal agranular insula	Contralateral caudal IPL
Ipsilateral dorsal dysgranular insula	
Ipsilateral rostroventral cingulate gyrus	
Ipsilateral cerebellum	
Contralateral lateral orbital gyrus	
Contralateral rostroventral fusiform gyrus	
Contralateral medioventral fusiform gyrus	
Contralateral lateroventral fusiform gyrus	
Contralateral rostrodorsal IPL	
Contralateral postcentral gyrus (trunk region)	
Contralateral caudodorsal cingulate gyrus	

Table S4. Changes in the nodal betweenness centrality when comparing OIE to TLE patients. Comparisons were performed using two-tailed t-tests. Significance was thresholded at $p<0.05$ uncorrected. PHG = parahippocampal gyrus; IPL = inferior parietal lobule; IFG = inferior frontal gyrus.

Increased clustering in patients with OIE	Increased clustering in patients with TLE
Ipsilateral dorsal IFG	Ipsilateral medioventral fusiform gyrus
Ipsilateral rostral IFG	Ipsilateral lateroventral fusiform gyrus
Ipsilateral medial orbital gyrus	Ipsilateral lateral posterior PHG
Ipsilateral caudal dorsolateral precentral gyrus	Ipsilateral rostrodorsal IPL
Ipsilateral precentral gyrus (upper limb region)	Ipsilateral ventral cingulate gyrus
Ipsilateral caudolateral ITG	Contralateral orbital area of the orbital gyrus
Ipsilateral rostroventral IPL	Contralateral lateral orbital gyrus
Ipsilateral lateral superior occipital gyrus	Contralateral caudal MTG
Ipsilateral dorsolateral putamen	Contralateral rostroventral fusiform gyrus
Ipsilateral medial prefrontal thalamus	Contralateral medioventral fusiform gyrus
Ipsilateral sensory thalamus	Contralateral lateroventral fusiform gyrus
Ipsilateral posterior parietal thalamus	Contralateral rostrodorsal IPL
Contralateral lateral SFG	Contralateral ventromedial putamen
Contralateral frontal operculum	
Contralateral caudal IPL	
Contralateral pregenual cingulate gyrus	
Contralateral caudal lingual gyrus	
Contralateral dorsal caudate nucleus	
Contralateral rostral temporal thalamus	

Table S5. Changes in regional clustering coefficients when comparing OIE to TLE patients. Comparisons were performed using two-tailed t-tests. Significance was thresholded at $p<0.05$ uncorrected. IFG = inferior frontal gyrus; ITG = inferior temporal gyrus; IPL= inferior parietal lobule; SFG= superior frontal gyrus; PHG = parahippocampal gyrus; MTG = middle temporal gyrus.

Increased local efficiency in patients with OIE	Increased local efficiency in patients with TLE
Ipsilateral dorsal IFG	Ipsilateral dorsolateral MTG
Ipsilateral medial orbital gyrus	Ipsilateral medioventral fusiform gyrus
Ipsilateral caudal dorsolateral precentral gyrus	Ipsilateral lateral posterior PHG
Ipsilateral precentral gyrus (upper limb region)	Ipsilateral rostrodorsal IPL
Ipsilateral caudolateral ITG	Ipsilateral ventral cingulate gyrus
Ipsilateral dorsolateral putamen	Contralateral frontal operculum
Ipsilateral medial prefrontal thalamus	Contralateral orbital area of the orbital gyrus
Contralateral caudal IPL	Contralateral lateral orbital gyrus
Contralateral caudal lingual gyrus	Contralateral caudal MTG
Contralateral ventromedial putamen	Contralateral rostroventral fusiform gyrus
Contralateral dorsal caudate nucleus	Contralateral medioventral fusiform gyrus
Contralateral rostral temporal thalamus	Contralateral lateroventral fusiform gyrus
	Contralateral lateral posterior PHG
	Contralateral pregenual cingulate gyrus

Table S6. Changes in the regional local efficiency when comparing OIE to TLE patients. Comparisons were performed using two-tailed t-tests. Significance was thresholded at $p<0.05$ uncorrected. IFG = inferior frontal gyrus; ITG = inferior temporal gyrus; IPL = inferior parietal lobule; MTG = middle temporal gyrus; PHG = parahippocampal gyrus.

CODE REPOSITORIES

Tractoflow version 2.2.0 (https://github.com/scilus/tractoflow/tree/2.2.0)

SCILPY library version 1.0.0 (https://github.com/scilus/scilpy/tree/1.0.0)

Surface-enhanced tractography version 1.1 (https://github.com/StongeEtienne/set nf/tree/v1.1.a)
